# Molecular Detection of Vector-Borne Pathogens and Their Association with Feline Immunodeficiency Virus and Feline Leukemia Virus in Cats from Northeastern Thailand

**DOI:** 10.3390/ani15142065

**Published:** 2025-07-12

**Authors:** Charinya So-In, Laksanachan Watayotha, Thikhamporn Sonsupee, Surasak Khankhum, Nuchsupha Sunthamala

**Affiliations:** 1 Department of Veterinary Technology, Faculty of Agricultural Technology, Kalasin University, Kalasin 46000, Thailand; charinya.pi@ksu.ac.th; 2 Department of Biology, Faculty of Science, Mahasarakham University, Mahasarakham 44150, Thailand; w.laksana0412@gmail.com (L.W.); pprawthikham@gmail.com (T.S.); surasak.kk@msu.ac.th (S.K.)

**Keywords:** domestic cats, feline immunodeficiency virus, feline leukemia virus, vector-borne pathogens, *Bartonella henselae*

## Abstract

In Thailand, domestic cats are frequently exposed to vectors that transmit a variety of pathogens. In this study, the prevalence of vector-borne pathogens (VBPs) and their association with feline leukemia virus (FeLV) and feline immunodeficiency virus (FIV) were investigated in 187 domestic cats from animal hospitals across five northeastern provinces. Twelve pathogens, including viruses, bacteria, and protozoa, were screened using PCR assays. FIV was identified in 2.67% of the cats, while FeLV exhibited a notably higher prevalence of 29.95%. Among the bacterial pathogens, *Bartonella henselae* was the most frequently detected (94.65%), followed by *Rickettsia felis* (34.22%). Protozoan infections such as *Babesia canis* (8.02%) and *Cytauxzoon felis* (3.21%) were less prevalent. Co-infections were common, with numerous cats hosting multiple pathogens. Correlation analysis revealed moderate associations between FIV and *Babesia microti* (r = 0.43), *Babesia canis* (r = 0.35), and *Mycoplasma* spp. (r = 0.33), indicating potential co-infection or predisposition. Although significant differences in the total white blood cell counts were not observed, leukopenia was more commonly found in FeLV/FIV-positive cats. These findings indicate that a high burden of infection and co-infection exists in the feline population, supporting the need for expanded pathogen screening and enhanced vector control strategies.

## Simple Summary

In this study on domestic cats from northeastern Thailand, blood samples were tested for viruses, bacteria, and protozoan parasites transmitted by vectors such as fleas and ticks. Feline leukemia virus (FeLV) was detected more commonly than feline immunodeficiency virus (FIV). Bacterial infections, especially from *Bartonella henselae* and *Rickettsia felis*, were common, while protozoan infections were less frequent. Many cats had multiple infections, particularly those positive for FIV or FeLV, likely due to weakened immunity. Although the overall white blood cell counts were similar, lower counts were more frequent in FIV/FeLV-positive cats. Regular screening for co-infections is recommended to improve feline health and reduce disease transmission risks.

## 1. Introduction

Fleas, ticks, and flies are commonly found on domestic cats in Thailand. These ectoparasites potentially transmit viruses, bacteria, rickettsiae, protozoa, and other parasites that cause feline vector-borne pathogens (VBPs) infections [1]. Nonetheless, the clinical signs in cats have historically received less attention compared to the same condition in dogs, which can significantly impair feline health and welfare [2].

*Anaplasma platys (A. platys)*, a tick-borne bacterium, infects platelets and leads to thrombocytopenia. The clinical signs in cats are generally mild or subclinical, though, in some cases, it can cause bleeding disorders, fever, and lethargy [3]. *Anaplasma phagocytophilum* (*A. phagocytophilum*), another tick-borne bacterium that infects neutrophils, causes granulocytic anaplasmosis, presenting with fever, lethargy, joint pain, and anorexia in infected cats. Severe cases may lead to immune suppression and secondary infections [4]. *Bartonella henselae* (*B. henselae*) is recognized as the causative agent of the zoonotic cat scratch disease (CSD), also known as cat scratch fever. It is transmitted by a scratch or bite from an infected cat, especially kittens. The disease in humans is characterized by enlarged and sensitive lymph nodes, typically located next to the site of the scratch or bite, and, occasionally, a small, erythematous and elevated lesion or pustule may manifest at the site of the scratch. CSD is often moderate and self-limiting, but it may lead to more severe problems in certain individuals, particularly in those with compromised immune systems. The persistence of a pathogen in an animal’s bloodstream significantly influences its zoonotic risk, as it increases the likelihood of transmission to other species, including humans. A pathogen’s ability to persist in a host’s bloodstream, often without causing severe illness, can create opportunities for transmission to new hosts. This is particularly relevant for zoonotic diseases, where the pathogen circulates in an animal reservoir and can spill over to humans [5].

*Bartonella burgdorferi* (*B. burgdorferi*), the causative agent of Lyme disease, is transmitted by *Ixodes* ticks and rarely infects cats. When infection does occur, clinical signs include fever, lameness, and joint swelling, with potential chronic complications if left untreated [6].

*Mycoplasma* spp., including *Mycoplasma haemofelis* (*M. haemofelis*), are transmitted by fleas, ticks, or other cats through fighting and can lead to feline infectious anemia. Affected cats may exhibit lethargy, pale gums, jaundice, and weight loss due to red blood cell destruction; untreated severe cases can be fatal [7]. *Rickettsia felis* (*R. felis*), transmitted by fleas, causes flea-borne spotted fever. While often subclinical in cats, some may show fever, lethargy, and mild discomfort, and fleas facilitate its spread among cats and to humans [8]. Protozoa pathogens such as *Babesia canis* (*B. canis*), *Babesia microti* (*B. microti*), *Cytauxzoon felis* (*C. felis*), and *Hepatozoon felis* (*H. felis*) also pose health risks. *Babesia* spp. infect red blood cells, causing babesiosis, which, though rare in cats, can lead to fever, lethargy, and hemolytic anemia, with severe cases resulting in multi-organ failure [7,9,10,11,12,13]. *C. felis* causes cytauxzoonosis, a potentially fatal disease characterized by fever, lethargy, jaundice, and respiratory distress, as the parasite invades macrophages, obstructing blood vessels and causing organ failure if untreated [13]. *H. felis*, transmitted through the ingestion of infected ticks, causes muscle pain, fever, and weight loss due to tissue invasion, leading to chronic, debilitating illness requiring long-term management [10,14].

Research on tick- and flea-borne diseases has not yet been widely undertaken, particularly in developing nations. VBPs play a significant role in transmitting diseases to cats, with fleas and ticks recognized as the main vectors. In Thailand, a myriad of stray and domesticated cats are allowed to wander the streets, public areas, and Buddhist monasteries [15]. This not only poses a risk to human health but also to other cats, as infected animals encountered outdoors may bring the infection into the home [16]. The presence of a large population of ownerless cats not only increases the transmission of VBPs among stray cats but also facilitates the spread of other infectious diseases through direct or indirect contact, particularly FeLV and FIV [16].

FIV, a retrovirus related to human immunodeficiency virus (HIV), primarily targets feline immune cells, particularly CD4+ T cells, resulting in immunosuppression. Transmission typically occurs through bite wounds during aggressive encounters, making outdoor male cats particularly vulnerable. The infection progresses through an acute phase characterized by mild fever and lymphadenopathy, followed by an asymptomatic phase, ultimately leading to feline-acquired immunodeficiency syndrome. In advanced stages, affected cats become highly susceptible to secondary infections and neoplasia [17,18]. Similarly, FeLV causes immunosuppression and can lead to anemia and lymphoma, spreading through close contact such as grooming or sharing food bowls [19]. Immunosuppression, particularly induced by FeLV and FIV, not only predisposes cats to VBP infections but also accelerates the appearance of clinical manifestations [1,17,20,21].

While many studies in Thailand have confirmed the prevalence of FIV/FeLV [17,22,23,24] and VBP [15,25,26,27,28,29] infections in Thailand, there is currently no information on FIV/FeLV and VBP co-infection in Thai household cats. This study aimed to assess the prevalence of FIV, FeLV, and VBPs in domestic cats from northeastern Thailand while investigating the correlations between these infections and alterations in white blood cell (WBC) populations, thereby enhancing our understanding of feline and zoonotic disease dynamics.

## 2. Materials and Methods

### 2.1. Ethical Approval

This research was conducted under the supervision of a licensed veterinarian and adhered to a proposal approved by the Institutional Animal Care and Use Committee at Mahasarakham University, Thailand (No. IACUC-MSU-55/2023).

### 2.2. Sample Collection

This study focused on domesticated and semi-domesticated cats residing in five provinces (Kalasin, Khon Kaen, Roi-et, Mahasarakham, and Udon Thani) in northeastern Thailand. In total, 187 cats were sampled between January and June 2023. Blood samples were collected from clinically healthy cats without clinical signs of infectious diseases. Blood samples (0.5–1.5 mL) from each cat were collected in ethylenediamine tetraacetic acid (EDTA)-coated vacuum vials (Greiner Bio-One, Kremsmünster, Austria) through the cephalic or saphenous vein. All collected samples were centrifuged (3000 rpm for 15 min) to separate red blood cells and plasma and kept at −20 °C until use for molecular investigation. Data, including age (animals ≤ 12 months of age were considered juvenile, while all others were considered adult) and sex (male or female), were collected through interviews with the caretakers in the monastery [30].

### 2.3. Blood Genomic DNA Extraction

Genomic DNA was extracted from blood samples using a GF-1 Blood DNA Extraction Kit (Vivantis, Subang Jaya, Selangor, Malaysia), following the manufacturer’s instructions [31,32]. Briefly, 200 µL of lysis buffer was added to 200 µL of blood in a microcentrifuge tube, and the mixture was thoroughly homogenized by pulsed vortexing. Next, 20 µL of Proteinase K was added, and the solution was mixed immediately. The mixture was incubated at 65 °C for 10 min. After incubation, 200 µL of absolute ethanol was added, followed by immediate and thorough mixing to ensure homogeneity. The sample was then transferred to the column, assembled with a clean collection tube, and centrifuged at 5000× *g* for 1 min, after which the flow-through was discarded. The column was washed twice with 500 µL of wash buffer, each followed by centrifugation at 5000× *g* for 1 min, with the flow-through discarded after each wash. To ensure purity maximum a final wash was performed with 500 µL of wash buffer, followed by centrifugation at maximum speed for 3 min. The column was then placed in a clean microcentrifuge tube, and 100 µL of preheated elution buffer was added directly onto the membrane. After standing for 2 min, the DNA was eluted by centrifuging at 5000× *g* for 1 min. The extracted DNA was stored at −20 °C. The ratio of absorbance at 260 and 280 nm was used to evaluate DNA quality, with values between 1.7 and 1.9 indicating acceptable purity for DNA [29].

### 2.4. Polymerase Chain Reaction (PCR) and Agarose Gel Electrophoresis

The extracted DNA served as a template for pathogen detection targeting the proviral DNA of FIV and FeLV as well as the genomic DNA from various vector-borne pathogens. Specific primers were utilized (Table 1) to detect FIV, FeLV, as well as several bacterial and protozoal pathogens, including *A. platys*, *A. phagocytophilum*, *B. henselae*, *B. burgdorferi*, hemotropic *Mycoplasma* spp., *R. felis*, *B. canis*, *B. microti*, *C. felis*, and *H. felis*. The PCR reaction mixture was prepared to a total volume of 25 µL, comprising 2 µL of DNA, 10X PCR buffer, 50 mM MgCl_2_, forward and reverse primers (10 µM each), 10 mM dNTPs, and 1 unit of Taq DNA polymerase (Vivantis, Subang Jaya, Selangor, Malaysia). The thermal cycling conditions included an initial denaturation at 95 °C for 5 min, followed by 40 cycles consisting of denaturation at 95 °C for 15 s, annealing at a time and temperature suitable for each primer (Table 1), and extension at 72 °C for 40 s, concluding with a final extension at 72 °C for 7 min. The PCR products were separated on 1.5% agarose gel (Vivantis, Subang Jaya, Selangor, Malaysia), stained with Red Safe (Vivantis, Subang Jaya, Selangor, Malaysia), and visualized under UV illumination to confirm the presence of the products at specific sizes (Table 1). Positive controls included DNA from serologically confirmed FIV- and FeLV-positive feline blood samples [32]. The positive results for FIV and FeLV were also confirmed using an Anigen Rapid FIV Ab/FeLV Ag Test (Woodley Equipment Company, Bolton, UK), which detects the feline immunodeficiency virus antibody and the feline leukemia virus antigen. The template DNA from reference bacterial and protozoan species was used as a positive control. A negative control was included in each round of PCR by substituting molecular-grade water (Hyclone, Logan, UT, USA) for the DNA template in the reaction mixture.

### 2.5. Blood Smear and Microscopic Examination

Thin blood smears were prepared from EDTA-treated blood samples and spread onto glass slides. The slides were air-dried and stained using the Wright–Giemsa staining dip method (Sigma, Carlsbad, CA, USA). Each thoroughly dried blood film was immersed feather-edge down in Wright–Giemsa stain for approximately 30 s before being placed in phosphate buffer (pH 6.8–7.2) for 1–10 min. The slides were then briefly rinsed in running deionized water and thoroughly air-dried prior to examination [43]. White blood cells were examined under a light microscope at 1250× magnification in a random sequence. At least 200 white blood cells in each sample were classified. The evaluation of cells and their sizes based on morphologic characteristics was described previously, such as for leukopenia (WBC < 5.5 × 10^3^/µL) and leukocytosis (WBC > 14 × 10^3^/µL) [15,44].

### 2.6. Statistical Analysis

Correlation analyses were performed using Spearman’s correlation coefficient, with a two-tailed significance level of *p* ≤ 0.05 considered statistically significant. Correlation coefficients were interpreted descriptively based on a commonly used classification: negligible (0.00–0.10), weak (0.10–0.39), moderate (0.40–0.69), strong (0.70–0.89), and very strong (≥0.90) [45]. We acknowledge that such thresholds may vary across disciplines and are not absolute. The differences in WBC counts were analyzed using a two-tailed unpaired *t*-test, with *p* ≤ 0.05 indicating significance. All statistical analyses were conducted using GraphPad Prism software version 10.

## 3. Results

### 3.1. Prevalence of FIV, FeLV, and VBPs in Domestic Cats

**Figure 1:**
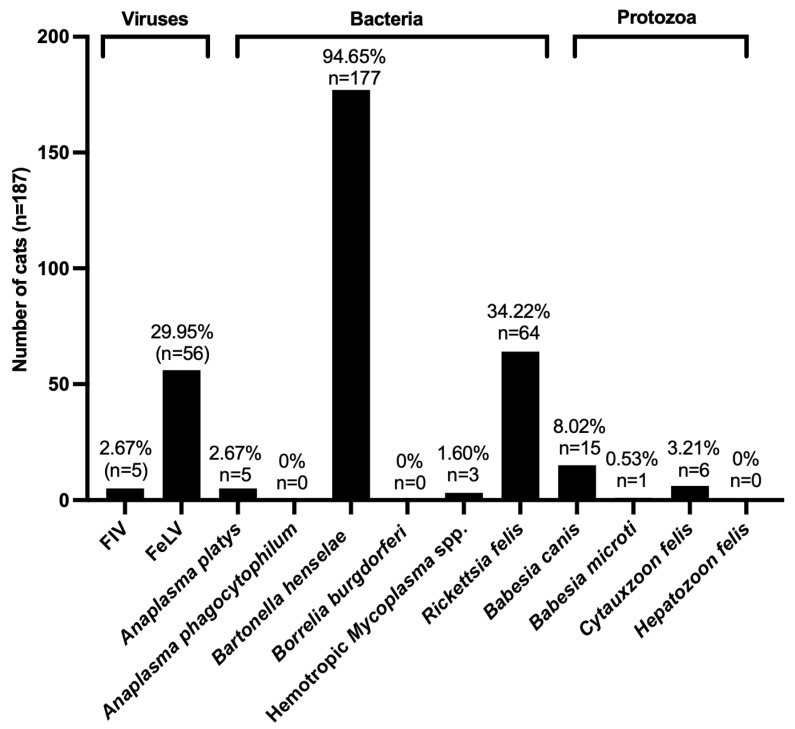
The prevalence and distribution of VBPs and infection patterns in domestic cats from northeastern Thailand (n = 187). The frequency of viral (FIV and FeLV), bacterial (*A. platys*, *A. phagocytophilum*, *B. henselae*, *B. burgdorferi*, hemotropic *Mycoplasma* spp., and *R. felis*), and protozoan *(B. canis*, *B. microti*, *C. felis*, and *H. felis*) infections identified using PCR. The percentages and number of infected cats (*n*) for each category are shown above the bars.

In total, 187 domestic cats were tested for FIV, FeLV, and VBPs using PCR. Among the viral pathogens, FIV was identified in five cats (2.67%), while FeLV exhibited a significantly higher prevalence, affecting 56 cats (29.95%). The bacterial pathogens presented a diverse range of detection rates, with *B. henselae* being the most prevalent, identified in 177 cases (94.65%). The other bacterial infections included *R. felis*, found in 64 cats (34.22%), and *A. platys* was detected in five cats (2.67%). No cases of *A. phagocytophilum* or *B. burgdorferi* were detected. *Mycoplasma* spp. were identified in only three cats (1.60%). Among the protozoan pathogens, *B. canis* was detected in 15 cats (8.02%), while *C. felis* and *B. microti* were found in six (3.21%) and one cat (0.53%), respectively. Importantly, *H. felis* was not found in any of the sampled cats. The notably high prevalence of *B. henselae* indicates its significance as a bacterial pathogen in domestic cats, while the prevalence rates of FeLV and *R. felis* emphasize the necessity for vigilant monitoring of both viral and bacterial infections within this population (Figure 1 and Figure S1).

### 3.2. Pattern of Infection of FIV, FeLV, and VBPs in Domestic Cats

**Figure 2:**
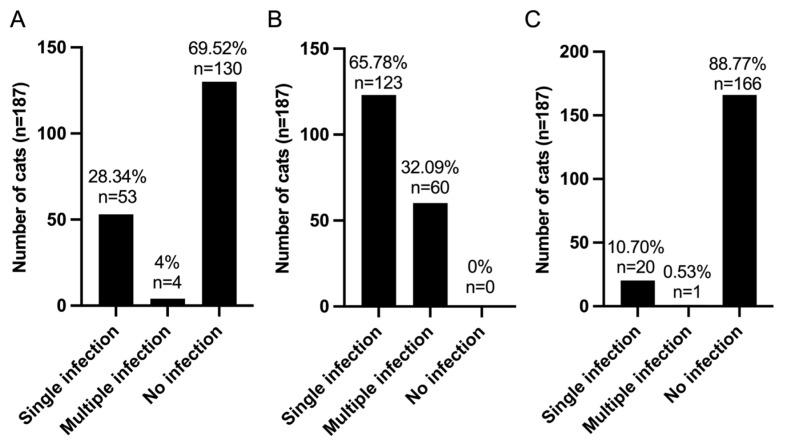
Infection patterns of FIV, FeLV, and VBPs in domestic cats from northeastern Thailand (*n* = 187). (**A**) Distribution of the viral infections; (**B**) distribution of bacterial infections; (**C**) distribution of protozoan infections. Single infection indicates monomicrobial infection, multiple infection indicates a pathogen co-infection with more than one pathogen, and no infection indicates absence of detection. Percentages and the number of infected cats (*n*) are displayed above each bar.

The infection patterns observed among the sample of 187 domestic cats were categorized based on the presence of viral, bacterial, and protozoan infections. In terms of viral infections, 53 cats (28.34%) exhibited a single viral infection, while four cats (2.14%) had multiple viral infections. A substantial proportion of the sample, comprising 130 cats (69.52%), showed no evidence of viral infection (Figure 2A). Conversely, bacterial infections were significantly more prevalent; 123 cats (65.78%) experienced a single bacterial infection, while 60 cats (32.09%) had multiple bacterial infections. Notably, no individuals in this cohort were free from bacterial infections (Figure 2B). Twenty cats (10.70%) were identified as having single protozoan infections, and only one cat (0.53%) had multiple protozoan infections involving both *B. canis* and *B. microti*. A large majority of the sample (88.77% or 166 cats) did not present with protozoan infections (Figure 2C).

These findings emphasize the considerable burden posed by bacterial infections within this population, characterized by a substantial prevalence of co-infections, while viral and protozoan infections were relatively infrequent.

### 3.3. Correlation Analysis of FIV, FeLV, and VBPs in Domestic Cats

**Figure 3:**
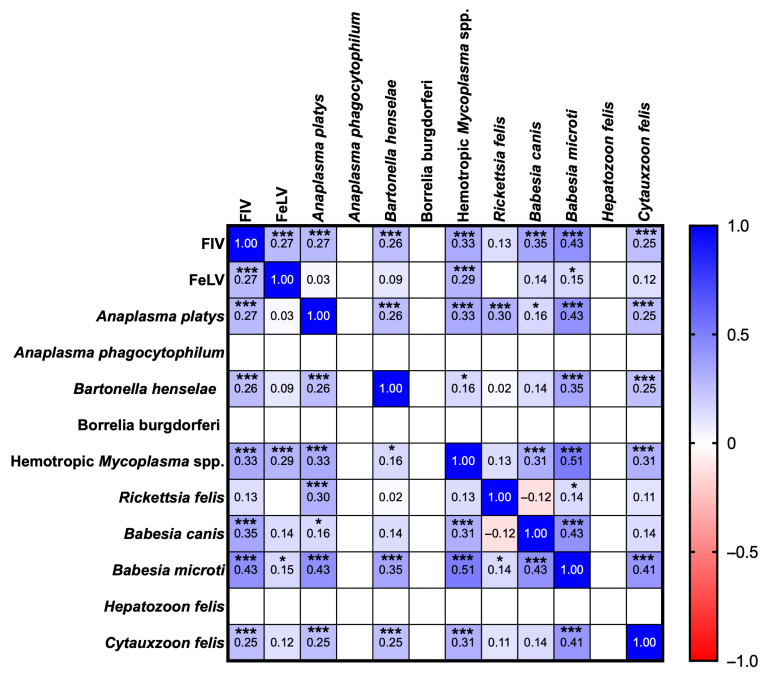
Correlation analysis of FIV, FeLV, and vector-borne pathogens in domestic cats. * *p* < 0.05, and *** *p* < 0.001.

Correlation analysis using Spearman’s coefficient indicated relationships among various pathogens within the sampled population. Specifically for viral pathogens, FIV demonstrated a moderate positive correlation with *B. microti* (r = 0.43, *p* = 0.0000) and weak correlations with *B. canis* (r = 0.35, *p* = 0.0000) and hemotropic *Mycoplasma* spp. (r = 0.33, *p* = 0.0000). These correlations suggest co-infection scenarios or underlying conditions that predispose infected cats to both FIV and these pathogens. Additionally, FIV exhibited weak correlations with FeLV (r = 0.27, *p* = 0.0002), *A. platys* (r = 0.27, *p* = 0.0001), *B. henselae* (r = 0.26, *p* = 0.0004), and *C. felis* (r = 0.25, *p* = 0.0004). In contrast, FeLV displayed low-to-negligible correlations with most pathogens; however, it showed a weak correlation with hemotropic *Mycoplasma* spp. (r = 0.29, *p* = 0.0001), followed by FIV (r = 0.27, *p* = 0.0000) and *B. microti* (r = 0.15, *p* = 0.0362), indicating possible co-infection dynamics. Among the bacterial pathogens, *A. platys* exhibited a moderate correlation with *B. microti* (r = 0.43, *p* = 0.0000) and weak associations with hemotropic *Mycoplasma* spp. (r = 0.33, *p* = 0.0000), *R. felis* (r = 0.30, *p* = 0.0000), FIV (r = 0.27, *p* = 0.0001), and *B. henselae* (r = 0.26, *p* = 0.0004). Meanwhile, *B. henselae* demonstrated a weak correlation with *B. microti* (r = 0.35, *p* = 0.0000), as well as with FIV and other pathogens, with r ranging from 0.25 to 0.26. Hemotropic *Mycoplasma* spp., on the other hand, exhibited a moderate positive association with *B. microti* (r = 0.51, *p* = 0.0000) and weak correlations with FIV (r = 0.33, *p* = 0.0000), *A. platys* (r = 0.33, *p* = 0.0000), and both *B. canis* and *C. felis* (r = 0.31, *p* = 0.0000). In contrast, *R. felis* displayed weak-to-negligible associations with the other pathogens within the matrix; a weak correlation was noted with *A. platys* (r = 0.30, *p* = 0.0000). Notably, *B. canis* correlated weakly with FIV (r = 0.35, *p* = 0.0000) and moderately with *B. microti* (r = 0.43, *p* = 0.0000). The observations regarding *B. microti* indicate some moderate correlations within the matrix, particularly with hemotropic *Mycoplasma* spp., suggesting frequent co-occurrence patterns among these pathogens (Figure 3).

### 3.4. Prevalence of VBPs in FIV/FeLV-Positive and FIV/FeLV-Negative Groups

**Figure 4:**
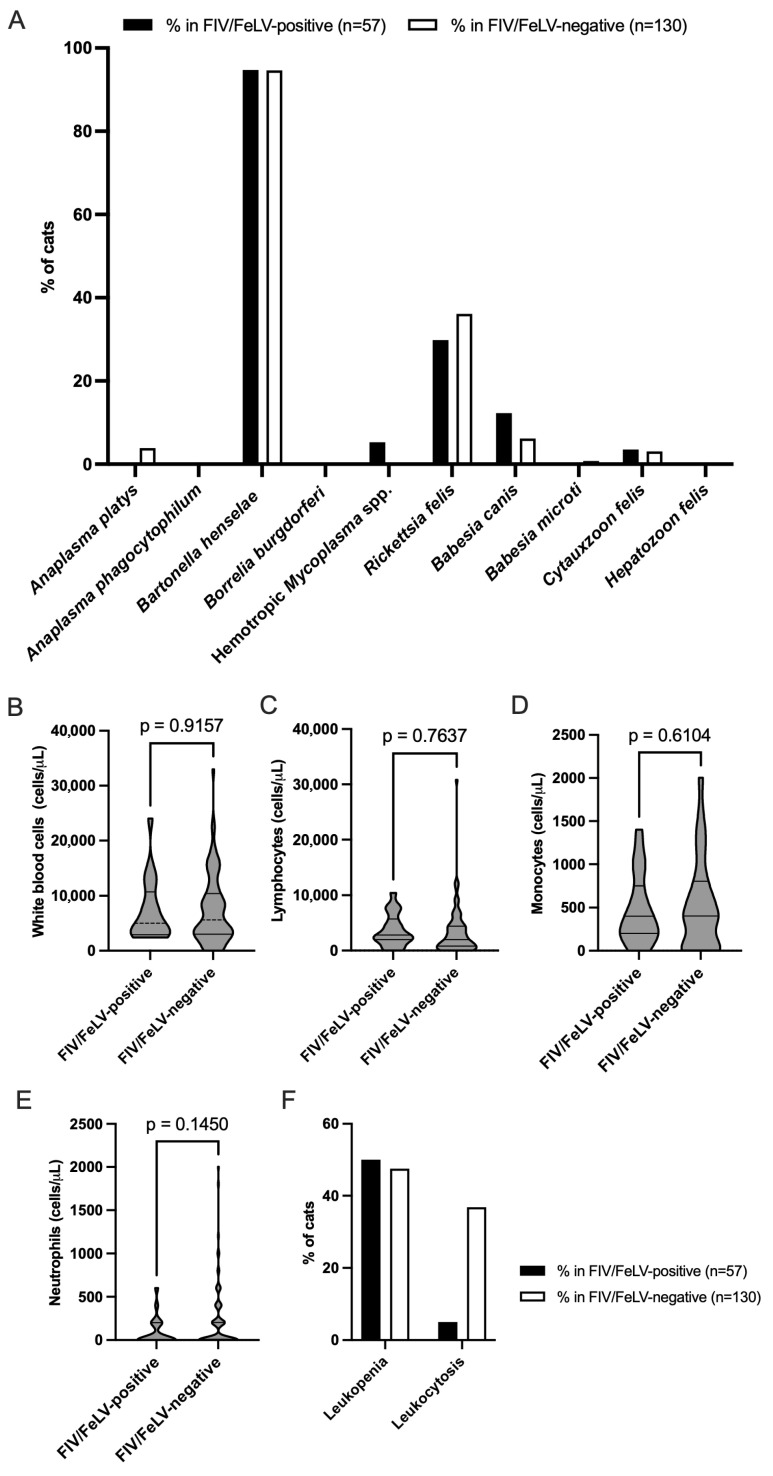
Prevalence of bacterial and protozoan pathogens, white blood cell counts, and their association with FIV/FeLV infection in domestic cats. (**A**) Prevalence of bacterial and protozoan pathogens in FIV/FeLV infections. (**B**) White blood cell count, (**C**) lymphocyte count, (**D**) monocyte count, and (**E**) neutrophil count in FIV/FeLV infections. (**F**) Percentage of cats with leukopenia and leukocytosis during FIV/FeLV infections.

Among the subset of samples positive for FIV/FeLV infections (57 cats), *B. henselae* was found to have the highest prevalence at approximately 94.74%, a figure closely mirroring its prevalence in the FIV/FeLV-negative cohort at approximately 94.62%. Conversely, infections caused by *A. platys* and *A. phagocytophilum* were absent in the FIV/FeLV-positive group, while *A. phagocytophilum* was present at a rate of 3.85% in the negative group. Hemotropic *Mycoplasma* spp., detected in approximately 5.26% of FIV/FeLV-positive cases, were absent from the negative group entirely. Additionally, *R. felis* was found in approximately 29.82% of the positive samples but exhibited an even higher prevalence among the negative samples at approximately 36.15%. Among the protozoal infections, *B. canis* was more prevalent in the FIV/FeLV-positive cases at approximately 12.28% compared to approximately 6.15% in the negative cases. *C. felis* had a small yet notable presence in both groups, 3.51% in the positive group versus 3.08% in the negative group, while *B. microti* appeared in just one negative sample. Both *B. burgdorferi* and *H. felis* were completely absent from both groups (Figure 4A).

### 3.5. White Blood Cell Counts and Their Association with FIV/FeLV Infections in Domestic Cats

Figure 4A exhibits the prevalence of various VBPs in domestic cats, comparing two groups: FIV/FeLV-positive cats (n = 57) and FIV/FeLV-negative cats (n = 130). Regardless of FIV/FeLV status, *B. henselae* was widely prevalent, as evidenced by its almost full frequency (nearly 100% prevalence) in both populations. *R. felis* prevalence was greater in the FIV/FeLV-negative cats compared to the FIV/FeLV-positive cats. *B. canis* was more prevalent in the FIV/FeLV-positive cats compared to the negative cats. Hemotropic *Mycoplasma* spp. were identified exclusively in the FIV/FeLV-positive cats but were absent in the negative cases. Regarding the other pathogens (e.g., *Anaplasma* spp., *B. burgdorferi*, *B. microti*, *C. felis*, and *H. felis*), the incidence in both groups varied from negligible to nonexistent. The analysis of white blood cell counts revealed no significant differences between the FIV/FeLV-positive and FIV/FeLV-negative groups concerning total white blood cell (Figure 4B), lymphocyte (Figure 4C), monocyte (Figure 4D), or neutrophil (Figure 4E) levels observed during the examination periods.

Leukopenia was common in both groups but slightly more frequent in the immunosuppressed cats (FIV/FeLV+), consistent with the immunosuppressive nature of these viruses. This result was found in approximately half (50%) of the FIV/FeLV-positive cats compared to approximately 47.5% among those classified as negative for these viruses. However, leukocytosis may be a response to infection in cats with an intact immune system. Its rarity in the FIV/FeLV-positive cats may reflect impaired immune responsiveness due to viral immunosuppression. This result is supported in Figure 4F: leukocytosis was observed much less frequently, at only 5% within the positive cases versus 36.84% among the negative cases.

This finding highlights the differential distribution of vector-borne pathogens and immune cell profiles in domestic cats based on their FIV/FeLV infection status. While *B. henselae* was highly prevalent regardless of viral status, certain pathogens such as *B. canis* and hemotropic *Mycoplasma* spp. were more associated with immunosuppressed cats. Additionally, the reduced frequency of leukocytosis in FIV/FeLV-positive cats suggests compromised immune responsiveness due to viral-induced immunosuppression.

## 4. Discussion

In the present study, the infection rates for FIV and FeLV were 2.67% and 29.95%, respectively, in northeastern Thailand. In a similar study conducted in another part of Thailand, the prevalence rates of FIV and FeLV infection were 3.5–16.5% and 0.4–16.5% between 2015 and 2022, respectively [17,22,23,24]. A study conducted in Bangkok and the surrounding areas from April 2013 to March 2014 reported an FIV prevalence of 5.4% among 777 sampled cats, with FeLV detected in 16.5% and co-infections observed in 3.5% of the cases. Infections were identified year-round, with FeLV being more prevalent and widely distributed across the region [22]. Another investigation involving 480 cats from Bangkok revealed that 12.5% were FeLV-positive, 8.3% were FIV-positive, and 2.7% had co-infections [17]. These findings are consistent with previous studies indicating that FeLV typically exhibits greater prevalence than FIV within domestic cat populations. This discrepancy may be attributed to the modes of transmission, as FeLV is often spread through social interactions such as grooming and sharing resources, which are common in multi-cat households [46]. In contrast, the relatively low prevalence of FIV supports the notion that its transmission primarily occurs through bite wounds, thereby limiting its spread in stable indoor populations [47,48]. Despite its lower prevalence, FIV carries considerable clinical implications; infected cats frequently exhibit immunosuppression, rendering them susceptible to opportunistic infections [47,49,50].

Additionally, VBPs showed a wide variety of detection rates in this research, with *B. henselae* being the most prevalent, identified in 94.65% of the cases. Analogous research was undertaken in Bangkok, Thailand. A separate study examining VBP prevalence among cats found that 63.7% were infected, with the predominant infections being *Babesia* spp. (39.5%) and hemoplasmas (36.9%). Male cats and those over one year old demonstrated higher infection rates, with significant hematological alterations noted, particularly macrocytic hypochromic anemia in cats infected with hemoplasmas. These findings demonstrate the necessity of ongoing surveillance of VBPs in Bangkok to identify potential vectors and implement strategies to reduce transmission among domestic cat populations [15].

Among the bacterial pathogens, *B. henselae* was detected at a notably high prevalence of 94.65%, reflecting its widespread presence in domestic cats, likely due to exposure to fleas, which serve as the primary vectors for *Bartonella* spp. This high prevalence raises public health concerns, since humans can contract bartonellosis from infected cats through scratches or bites [27,51]. In Thailand, the average prevalence of *Bartonella* spp. infection among stray and pet cats is reported at 27.6% across nine regions, with notable rates reported in Khon Kaen (50.1%), Roi Et (36.8%), Ratchaburi (34.8%), Chiang Mai (23.3%), Kanchanaburi (21.4%), Nakhon Ratchasima (20%), Songkhla (12.8%), Bangkok (5.6%), and Ubon Ratchathani (11.8%) [25,28]. Human seroprevalence for *Bartonella* spp. has been recorded at approximately 5.5%, with occasional detections using culture and PCR methods, suggesting the need for increased awareness regarding *Bartonella* spp. risks in Thailand and Asia [5,52,53]. In a study analyzing blood samples from 139 cats across four Thai provinces between January 2014 and January 2015, a total of 9.4% tested positive for various *Bartonella* species, including *B. henselae*, *B. clarridgeiae*, and *B. vinsonii* subsp. *Berkhoffii* [26]. The high incidence of *B. henselae* in this study might be attributed to a variety of causes, including the high density of stray cats in Thailand’s northeastern region, which leads to substandard healthcare for these homeless cats, resulting in some asymptomatic carriers of several diseases [25,28]. The tropical climate and high humidity in Thailand may contribute to sustained transmission from the increase in external parasites, including fleas and flea activity, leading to potential contact with *B. henselae*-infected animals [54].

Other bacterial pathogens, such as *R. felis* (34.22%) and *A. platys* (2.67%), were also detected but at lower rates than *B. henselae*. *R. felis*, another flea-borne pathogen, has garnered attention as a cause of flea-borne spotted fever in humans; therefore, its detection in feline populations is significant for both veterinary and public health [8,55]. In a separate study involving 167 symptomatic cats at veterinary clinics in Tekirdağ, Turkey, where PCR was utilized to rapidly diagnose 14 pathogens, *B. henselae* exhibited the highest prevalence at 40.1%, followed by *A. platys* at 30.5% and *R. felis* at 26.3%. Conversely, *B. microti* was found at a much lower rate of 2.4% [56].

*Mycoplasma* spp. were present in only 1.60% of cases. In the protozoan category, *B. canis* was identified in 8.02%, while *C. felis* and *B. microti* were detected in 3.21% and 0.53%, respectively. Importantly, *H. felis* was not found in any of the sampled cats. This represents a relatively low prevalence compared to *B. henselae*. However, both pathogens are clinically significant due to their potential to cause hemolytic anemia in cats, particularly those with underlying immunosuppressive conditions such as FIV or FeLV [15]. The absence of *A. phagocytophilum* and *B. burgdorferi* suggests the limited exposure to these pathogens within the study population, possibly due to geographical or environmental factors influencing tick vector distribution. Protozoan infections were less prevalent. *B. canis* was identified as the most common at a rate of 8.02%, followed by *C. felis* at 3.21% and *B. microti* at just 0.53%. The low detection rates for these protozoan pathogens indicate limited vector exposure or low transmission rates within the studied population; *Babesia* spp. infections are generally associated with tick exposure, which may be less prevalent among indoor or urban-dwelling cats [9]. Although *C. felis* is infrequently encountered in domestic cat populations, it is recognized as a severe pathogen associated with high mortality rates in infected felines; its primary mode of transmission is also through ticks, particularly prevalent in North America. The absence of *H. felis* within this cohort suggests either its limited distribution or rarity within the region under investigation. These findings align with previous research that has identified *Babesia* spp. and *Cytauxzoon* spp. as emerging threats to feline health in regions characterized by high tick activity [57,58].

This study further explored the co-infection patterns and correlations among various pathogens, revealing that bacterial infections were more prevalent than viral or protozoan infections within this population. A significant proportion of cats (32.09%) presented with multiple bacterial infections, indicating a considerable bacterial burden among the domestic cats studied. Co-infections involving multiple bacterial species, particularly between *B. henselae* and *R. felis*, likely reflect shared vectors and environmental conditions conducive to flea infestations [55]. Correlation analyses indicated moderate associations between FIV and several pathogens, including *B. microti*, *B. canis*, and *Mycoplasma* spp., suggesting that immunosuppression induced by FIV may increase susceptibility to these infections. Weak correlations were also noted between FeLV and certain bacterial pathogens; this finding indicates an elevated risk for co-infections among FeLV-positive cats due to compromised immune function [17,55,59]. The classification of correlation strength remains a topic of debate and may vary by context. Therefore, the interpretations presented here should be viewed as descriptive rather than definitive.

Additionally, a moderate correlation was identified between hemotropic *Mycoplasma* spp. and *B. microti* (r = 0.51, *p* = 0.0000), suggesting shared vectors or predisposing factors for co-infection scenarios. Further analysis comparing pathogen prevalence between FIV/FeLV-positive and FIV/FeLV-negative groups revealed that *B. henselae* maintained a high prevalence in both groups, suggesting a transmission mechanism independent of viral status. In contrast, pathogens such as *Mycoplasma* spp. and *B. canis* were more frequently observed in the FIV/FeLV-positive cats. This supports the hypothesis that immunosuppression predisposes these individuals to increased susceptibility toward VBPs infections [15]. Conversely, *R. felis* exhibited greater prevalence in the FIV/FeLV-negative cats; this observation indicates influences unrelated to viral immunosuppression, such as environmental exposure factors linked specifically to flea infestations. White blood cell analysis revealed higher rates of leukopenia among the FIV/FeLV-positive cats, consistent with the immunosuppressive effects attributed to these viral infections, which can inhibit leukocyte production and function effectively [17,60]. In contrast, leukocytosis was more prevalent among the FIV/FeLV-negative individuals; this finding suggests inflammatory or infectious processes occurring independently from viral-induced immunosuppression [15]. These findings emphasize the diagnostic value of leukocyte counts in differentiating immune status and identifying secondary infections in FIV/FeLV-infected cats.

## 5. Conclusions

This study demonstrated high prevalences of FIV and FeLV infections in northeastern Thailand, with FeLV exhibiting a greater prevalence. The greatest frequency of VBPs, including *B. henselae*, was seen in household cats. Flea-borne infections such as *R. felis* and *A. platys* were identified in a substantial percentage of the cats. *Babesia* spp. and *Cytauxzoon* spp. were recognized as emerging concerns. Bacterial infections were more common than viral or protozoan ones, with over 30% of the cats exhibiting multiple infections. The identified co-infection patterns indicate that immunosuppression from FIV and FeLV renders cats more susceptible to other infections, highlighting the necessity for thorough pathogen screening in FIV/FeLV-positive cats. Although a high detection rate of *B. henselae* was noted, confirmatory testing by sequencing or alternative assays was not performed and should be addressed in future investigations. These findings support the need for integrated pathogen surveillance and targeted health management strategies to improve feline welfare and reduce the risk of zoonotic transmission.

## Figures and Tables

**Table 1:** Specific primers for the detection of viruses, bacteria, and protozoa from the blood of domestic cats.

Pathogen Category	Organisms and Primers	Annealing Temperature (°C)	PCR Product Size (Base Pair)	References
Viruses	FIV FW: CAATGGCCATTAAATGAA RV: AGAGAGGCCTGGAATCAAAT FeLV FW: GAAAGTACACAAAAACAGGAG RV: CTTAAGTCCTGCACTGG	54	1137	[33]
		49	305	[33]
Bacteria	*Anaplasma platys* FW: GATTTTTGTCGTAGCTTGCTATG RV: TAGCACTCATCGTTTACAGC *Anaplasma phagocytophilum* FW: ATGAATTACAGAGAATTGCTTGTAGG RV: TTAATTGAAAGCAAATCTTGCTCCTATG *Bartonella henselae* FW: TTCCGYCTTATGGGTTTTGG RV: CATTTCTGTTGGAAATCCTAG *Borrelia burgdorferi* FW: AATAGGTCTAATATTAGCCTTAATAGC RV: TCAAGTCTGGTTCCGTCTGCTC Hemotropic *Mycoplasma* spp. FW: GCCCATATTCCTACGGGAAGCAGCAGT RV: CTCCACCACTTGTTCAGGTCCCCGTC	55	678	[34]
		54	846	[35]
		52	246	[36]
		60	417	[37]
		68	620	[38]
	*Rickettsia felis* FW: CCGATTCAGCAGGTTCTTCAA RV: ATGTTCGGGCTTCCGGTATG	57	120	[36]
Protozoa	*Babesia canis* FW: GTGAACCTTATCACTTAAAGG RV: CTACACAGAGCACACAGCC	56	746	[39]
	*Babesia microti* FW: ATAGGTCAGAAACTTGAATGATACA RV: CTTAGTATAAGCTTTTATACAGC *Cytauxzoon felis* FW: CCAGCTCCAATAGCGTATATT RV: AGGATGAACTCGATGAATGCA *Hepatozoon felis* FW: CTTACCGTGGCAGTGACGGT RV: TGTTATTTCTTGTCACTACCTCTCTTATGC	55	238	[40]
		61	431	[41]
		58	146	[42]

## Data Availability

All data generated or analyzed during this study are included in the article and are available from the corresponding author upon reasonable request.

## Supplementary Materials

The following supporting information is available at https://www.mdpi.com/article/10.3390/ani15142065/s1: Figure S1: Distribution of FIV, FeLV, and vector-borne pathogens in the tested cats. The heat map represents an individual cat, with blue indicating pathogen detection. Figure S2: Gel electrophoresis showing representative PCR products.
