# Supplementary material for: Molecular Detection of Vector-Borne Pathogens and Their Association with Feline Immunodeficiency Virus and Feline Leukemia Virus in Cats from Northeastern Thailand
Source: Animals (Basel). 2025 Jul 12;15(14):2065. doi: 10.3390/ani15142065 (PMC12291768; doi:10.3390/ani15142065)
Supplement: Supplementary file 1 [file animals-15-02065-s001.zip › animals-3640280-supplementary.pdf]

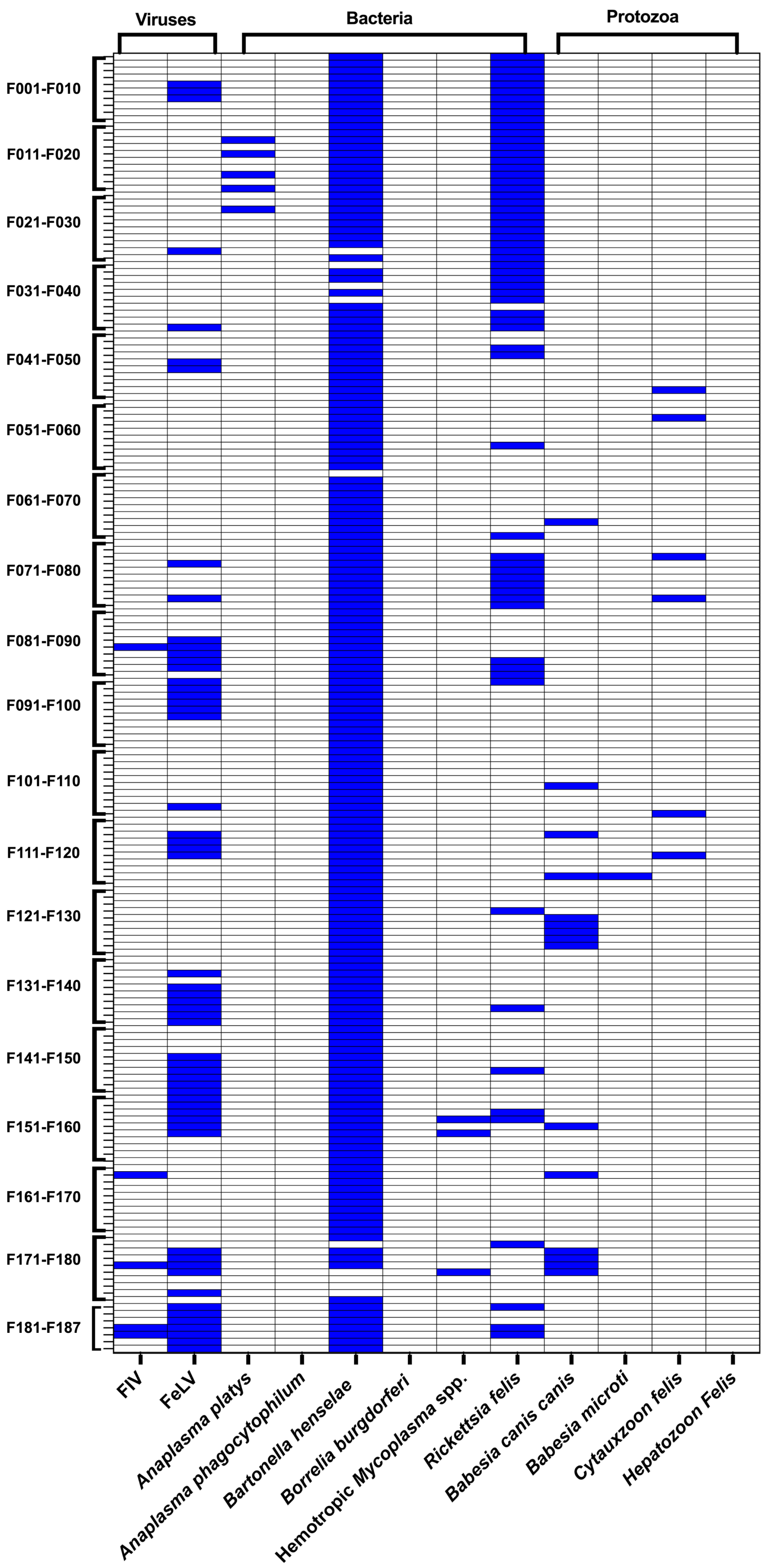

Supplementary Figure S1. Distribution of FIV, FeLV and vector-borne pathogens in tested cats. Heat maps represent individual cats. Blue indicated the presence of pathogens.

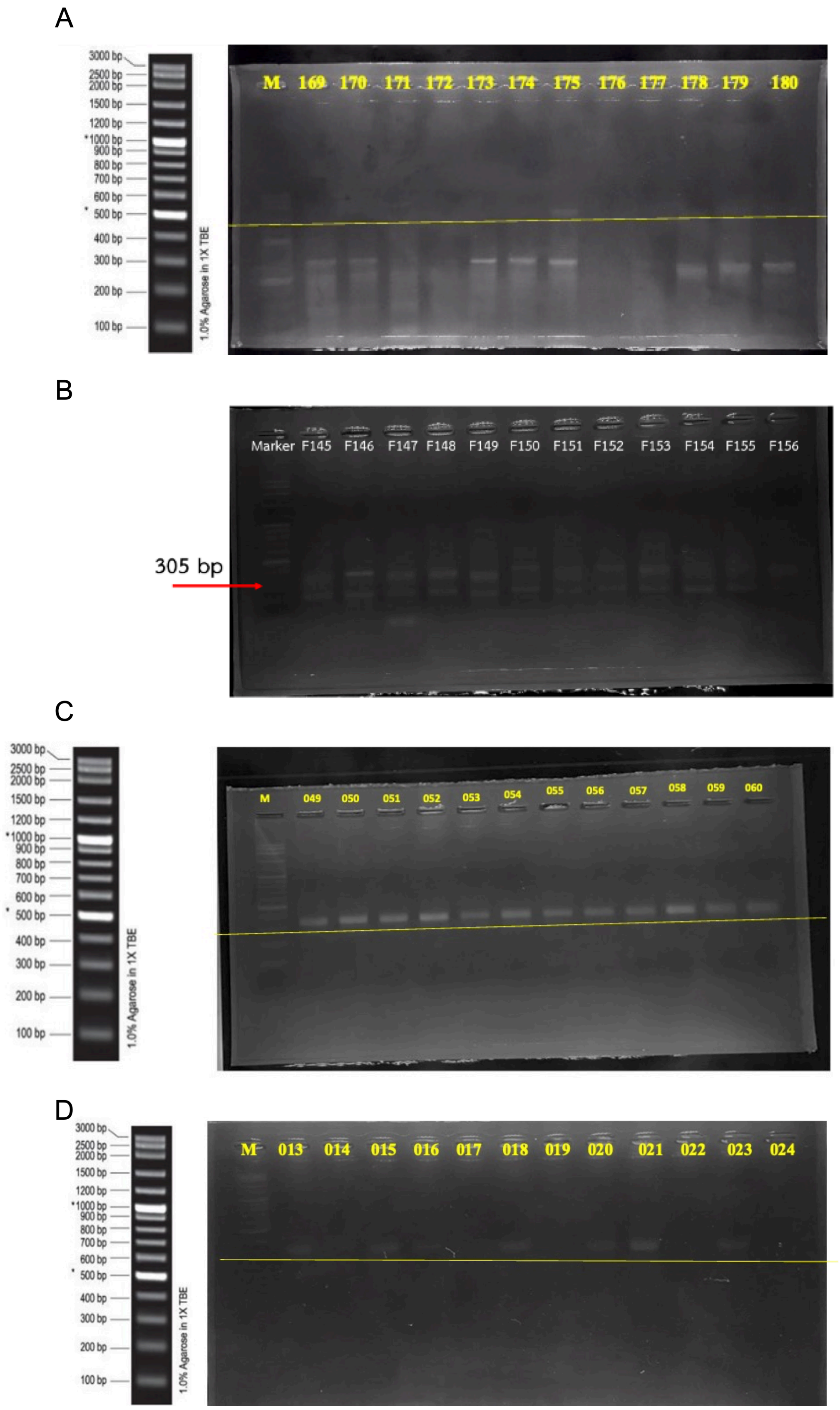

Supplementary Figure S2. Gel electrophoresis of representative PCR products. (A) Feline Immunodeficiency Virus (FIV) amplicon: 1137 bp; (B) Feline Leukemia Virus (FeLV) amplicon: 305 bp; (C) *Bartonella henselae* amplicon: 246 bp; (D) *Anaplasma platys* amplicon: 678 bp.
